# Impact of transitional care on endocrine and anthropometric parameters in Prader–Willi syndrome

**DOI:** 10.1530/EC-18-0089

**Published:** 2018-04-17

**Authors:** A C Paepegaey, M Coupaye, A Jaziri, F Ménesguen, B Dubern, M Polak, J M Oppert, M Tauber, G Pinto, C Poitou

**Affiliations:** 1 Nutrition DepartmentAssistance-Publique Hôpitaux de Paris (AP-HP), Pitié-Salpêtrière Hospital, French Reference Center for Prader-Willi Syndrome, Sorbonne Université, Paris, France; 2 Nutrition and Gastroenterology DepartmentAssistance-Publique Hôpitaux de Paris (AP-HP), Armand Trousseau Children’s Hospital, Paris, France; 3 Pediatric EndocrinologyDiabetology and Gynecology Department, Assistance-Publique Hôpitaux de Paris (AP-HP), Necker Enfants Malades Hospital University Hospital, Paris, France; 4 Pediatric Endocrinology DepartmentChildren’s Hospital, French Reference Center for Prader-Willi Syndrome, Toulouse, France; 5 INSERMUMRS 1166, Nutriomic Group 6, Paris, France; 6 Sorbonne UniversitéUMRS1166, Paris, France

**Keywords:** Prader–Willi syndrome, transition, GH treatment, obesity

## Abstract

**Context:**

The transition of patients with Prader–Willi syndrome (PWS) to adult life for medical care is challenging because of multiple comorbidities, including hormone deficiencies, obesity and cognitive and behavioral disabilities.

**Objective:**

To assess endocrine management, and metabolic and anthropometric parameters of PWS adults who received (*n* = 31) or not (*n* = 64) transitional care, defined as specialized pediatric care followed by a structured care pathway to a multidisciplinary adult team.

**Patients and study design:**

Hormonal and metabolic parameters were retrospectively recorded in 95 adults with PWS (mean ± s.d. age 24.7 ± 8.2 years, BMI: 39.8 ± 12.1 kg/m²) referred to our Reference Center and compared according to transition.

**Results:**

Among the entire cohort, 35.8% received growth hormone (GH) during childhood and 16.8% had a GH stimulation test after completion of growth. In adulthood, 14.7% were treated with GH, 56.8% received sex-hormone therapy, whereas 91.1% were hypogonadic and 37.9% had undergone valid screening of the corticotropic axis. The main reason for suboptimal endocrine management was marked behavioral disorders. Patients receiving transitional care were more likely to have had a GH stimulation test and hormonal substitutions in childhood. They also had a lower BMI, percentage of fat mass, improved metabolic parameters and fewer antidepressant treatments. Transitional care remained significantly associated with these parameters in multivariate analysis when adjusted on GH treatment.

**Conclusion:**

A coordinated care pathway with specialized pediatric care and transition to a multidisciplinary adult team accustomed to managing complex disability including psychiatric troubles are associated with a better health status in adults with PWS.

## Introduction

Prader–Willi syndrome (PWS) is a complex developmental genetic disorder including obesity with hyperphagia, psychiatric disturbances, endocrine and metabolic disorders (1, 2). Global incidence has been recently evaluated as 1 in 21,000 births in France (1). PWS is caused by the lack of expression of paternal-origin imprinted alleles on chromosome 15 (2). PWS is now usually diagnosed early in life and then managed by pediatric teams (1). The transition of patients with PWS from adolescence to adult life is challenging for medical care because of multiple comorbidities, behavioral problems, specific skills required of the medical team and lack of an established scheme for the change from pediatric to adult medical teams.

Endocrine disorders in PWS patients are part of a complex presentation including severe neonatal hypotonia and poor feeding skills, excessive weight gain at an early age and subsequent development of obesity with hyperphagia and lack of satiety in children, cognitive disabilities and psychiatric features (3). The most frequent endocrine involvement includes hypogonadism with cryptorchidism in 80% of cases and primary amenorrhea in 56% of cases (2). Growth hormone (GH) deficiency is present in 90% of children (4, 5). The prevalence of other endocrine disorders appears variable depending on the study: hypothyroidism ranges from 2 to 72% of cases (6, 7, 8) and central adrenal insufficiency from 7.5 to 60% of cases (9, 10). Many changes take place during adolescence and may specifically affect young patients with endocrine disorders, as they may have more difficulty integrating with their peers (11). In addition, teenagers with PWS are particularly vulnerable, because this transition time is characterized by the emergence or worsening of many comorbidities, such as hyperphagia, behavioral disorders, scoliosis (12) and obesity-related complications (11), together with a strong feeling of the differences between them and their peers. Excessive fat mass is a critical problem for PWS teenagers and young adults, as it is associated with severe complications, such as type 2 diabetes, cardiac or respiratory failure and physical disabilities (13). Behavioral disorders, including obsessiveness, temper outbursts, emotional lability and skin picking, often increase in late adolescence and early adulthood (14).

Adolescence is often associated with a loss of follow-up for all patients with chronic disease (15, 16). The transition from pediatric to adult health care is even more challenging in adolescents with PWS, especially because of cognitive and psychiatric disorders. Moreover, PWS is a rare disease characterized by a complex disability. Medical care may be poorly adapted to patients because of a lack of knowledge concerning the syndrome when they are not followed in a reference center. Thus, patients and caregivers face with psychological, medical and social issues that can lead to a break in the care pathway. No study has examined transitional care and its impact on adult health in PWS.

The aim of our study was to compare endocrine management and anthropometric and metabolic parameters of PWS patients, with or without optimal pediatric follow-up and transitional care.

## Patients and methods

### Patients

Between January 2007 and June 2017, 95 adults (age ≥ 16 years) with genetically confirmed PWS were referred to the French Reference Center with PWS, La Pitié-Salpêtrière Hospital, Paris, France and were examined. Ethical approval from the Research Ethics Committee of Hôtel-Dieu Hospital (CPP Ile de France 1) was obtained for the constitution of a database, and all patients and guardians/parents gave informed consent for this observational study.

The cohort was divided into two groups. The first group, named the ‘transition group’ (*n* = 31), included patients who had received specialized and multidisciplinary pediatric care including endocrine follow-up in one of six children hospitals with expertise in the follow-up of children with PWS. In late adolescence (16–19 years), these patients were referred to the adult unit of the Reference Center. The pediatrician then provided the medical file and transferred all medical data of the pediatric follow-up to the adult physician. A first multidisciplinary assessment was then carried out in the adult department during a day hospitalization. An endocrinologist with specialized nutrition training, a dietician, psychologist, nurse and social worker were all present for this first evaluation. The second group, named the ‘without transition’ group (*n* = 64), included patients who were referred to the Reference Center, not by a pediatric hospital team, but by their psychiatrist (*n* = 11), the Prader–Willi France national patient organization (*n* = 11), their endocrinologist (*n* = 9), the physician from a group home (*n* = 7), another adult hospital department (*n* = 6), their general practitioner (*n* = 5), a bariatric surgeon (*n* = 4), their parents or siblings (*n* = 4), a post-acute rehabilitation department (*n* = 3%), another physician (*n* = 3) or another patient with PWS (*n* = 1). This second group also underwent a multidisciplinary assessment during a day hospitalization.

The most frequent reasons for the absence of transitional care were disruption in medical follow-up during the pediatric period (*n* = 29, 45.3%), late diagnosis of PWS (21.6 ± 11.5 years) (*n* = 20, 31.3%), lack of specialized pediatric care since diagnosis (*n* = 13, 20.3%) or refusal of the proposed care pathway by the family (*n* = 2). The reasons of loss to follow-up during the pediatric period were a non-cooperative family (*n* = 13), lack of specialized pediatric care (*n* = 11), psychiatric disorders (*n* = 4) or distance from the Reference Center (*n* = 1).

### Clinical, anthropometric and biological assessments

All subjects underwent a standardized medical interview, a physical examination and fasting venous blood sample for routine biological analyses. Hormonal and metabolic biochemical parameters were measured using routine techniques, as previously described (17). The presence of diabetes, high blood pressure (HBP), dyslipidemia, respiratory and cardiac disorders, hormonal deficits and their respective treatments, including psychotropic medications were recorded and defined, as previously described (17).

Fat mass (FM) was measured by whole-body fan-beam dual energy x-ray absorptiometry (DXA, Hologic Discovery W, software v12.6; Hologic, Bedford, MA). The fat mass index (FMI), appendicular FM, trunk FM and the ratio of trunk FM (kg) to appendicular FM (kg) were calculated, as previously described (18).

### Patients and families questionnaire

A questionnaire with 28 questions concerning the point of view of the patient and his/her family on the social and medical pathway from childhood to adulthood was sent to 49 patients. The questionnaire was not sent to patients who were aged older than 50 years, lost to follow-up, did not speak French or those with a non-cooperative family. A total of 33 patients answered the questionnaire: 13 were in the transition group and 20 the without transition group.

### Statistical analysis

Categorical data are presented as numbers and percentages and continuous variables as the means ± s.d. and minimum and maximum. Nonparametric Wilcoxon and *X* ^2^ tests (or Fischer’s exact test for small samples) were used for the comparison of continuous or categorical variables, respectively. Multiple regression analyses were used to assess the independent associations of BMI, body composition and psychiatric treatments with transition status, adjusting for current age, age at first multidisciplinary assessment, gender, genotype, age at genetic diagnosis, sex-hormone treatment in adulthood, GH treatment during childhood and/or in adulthood. The two-sided significance level was fixed at 5%. Statistical analyses were performed using JMP statistic software (SAS Institute Inc. Cary, NC, USA).

## Results

### Endocrine parameters depending on transition status

There was no significant difference in gender or genotype depending on transition status. However, the genetic diagnosis was made earlier for the transition group: 5.7 ± 6.1 vs 14.9 ± 12.2 years of age (*P* < 0.001). Patients who received optimal pediatric care with transition were younger and had their first multidisciplinary assessment in the adult nutrition department earlier than patients without transitional care (Table 1).

**Table 1:** Endocrine status according to transition status from pediatric to adult care.

	All (*n* = 95)	Transition (*n* = 31)	Without transition (*n* = 64)	*P*
Age at diagnosis (year)	7.5 ± 8.0 (0.0; 40.0)	5.7 ± 6.1	14.9 ± 12.2	**<0.001**
Age at multidisciplinary assessments in adult nutrition department (year)	24.7 ± 8.2 (16.1; 58.7)	19.9 ± 3.1	27.1 ± 8.9	**<0.001**
Female gender (%)	51 (53.7)	17 (54.8)	34 (53.1)	0.88
Genotype				
Deletion (%)	50 (52.6)	17 (54.8)	33 (51.6)	0.58
Uniparental disomy (%)	39 (41.1)	14 (45.2)	25 (39.1)	
Imprinting defects (%)	2 (2.1)	0 (0)	2 (3.1)	
Somatotropic axis				
During childhood				
GH stimulation test (%)	44 (46.3)	23 (74.2)	21 (32.8)	**0.001**
GH deficiency (%)	41/44 (93.2)	20/23 (87.0)	21/21 (100)	0.99
GH treatment (%)	34 (35.8)	19 (61.3)	15 (23.4)	**<0.0001**
After completing growth				
GH stimulation test (%)	16 (16.8)	7 (22.6)	9 (14.1)	0.43
GH deficiency (%)	8/16 (50.0)	2/7 (28.6)	6/9 (66.7)	0.17
GH treatment (%)	14 (14.7)	8 (25.8)	6 (9.4)	0.08
Gonadotropic axis				
Before transition				
Sex-hormone therapy (%)	20 (21.1)	16 (51.6)	4 (6.3)	**<0.0001**
After transition				
Screening (%)^§^	90 (94.7)	30 (96.8)	60 (93.8)	1
Hypogonadism (%)	82/90 (91.1)	27/30 (90.0)	55/60 (91.7)	1
Sex-hormone therapy (%)	54 (56.8)	19 (61.3)	35 (54.7)	0.89
Corticotropic axis in adulthood				
Screening (%)*	36 (37.9)	12 (38.7)	24 (37.5)	1
Central adrenal insufficiency (%)	3/36 (8.3)	1/12 (8.3)	2/24 (8.3)	1
Thyrotropic axis in adulthood				
Screening (%)	91 (95.8)	31 (100.0)	60 (93.8)	0.59
Hypothyroidism (%)	29/91 (31.2)	10/31 (32.3)	19/60 (31.7)	0.99
l-Thyroxine treatment (%)	22 (23.2)	8 (25.8)	14 (21.9)	0.99

Categorical data are presented as numbers and percentages. Continuous variables are presented as the means ± s.d., minimum and maximum. Nonparametric Wilcoxon and *X* ^2^ tests (or Fischer’s exact test for small sample size) were used for comparisons of continuous or non-continuous variables, respectively.

*Cortisol level at 08:00 h <138 nmol/L or >500 nmol/L or dynamic test performed; ^§^measurement of estradiol or testosterone and gonadotrophins levels at baseline.

Among the entire cohort, less than half the patients had a stimulation test to assess the GH/IGF1 axis during childhood and only approximately one of six patients after they completed growing (Table 1). Two times more patients of the transition group had a GH stimulation test in childhood than those without transition. GH deficiency (GHD) was found in almost all of the children when performed in childhood before GH start and was found in half of the adults. Only about one-third of patients of the cohort received GH treatment during childhood, but two times more patients of the transition group had GH treatment than those without transition. This difference remained significant after exclusion of patients for whom the diagnosis of PWS was received after the age of 15 years (exclusion of 14 patients (14.7%), 61.5% vs 31.8%, *P* = 0.02). There was a trend toward more GH stimulation test after completion of growth in patients who received GH treatment in childhood (26.5% vs 11.5%, *P* = 0.06). Only approximately one of six adults received GH treatment, with a trend toward a higher percentage for the patients with transition.

We analyzed patients case by case to understand why most patients did not have a GH stimulation test after completion of growth (*n* = 79, 83.2%), although it is recommended by current guidelines (19, 20). The main reason (*n* = 35, 44.3%) was the presence of marked behavioral disorders. The other reasons were refusal to continue or start GH treatment (*n* = 18, 22.8%), uncontrolled diabetes (*n* = 8, 10.1%), another medical problem (*n* = 6, 7.6%), technical difficulties (*n* = 4), loss to follow-up soon after their first medical visit in the adult department (*n* = 4), social problems (*n* = 2), refusal to discontinue GH treatment (*n* = 1) or fear of weight gain after discontinuing GH treatment (*n* = 1).

Concerning patients who received GH treatment in childhood (*n* = 34), 11 subjects still received GH treatment in adulthood of which seven received it without a prior GH stimulation test for the above-listed same reasons. Five stopped treatment because of a normal GH stimulation test. Moreover, 18 patients discontinued GH without a prior GH stimulation test because of behavioral disorders for six patients, completion of growth for five, the wish of the family or patient for three or medical problems, such as glucose intolerance, uncontrolled diabetes, aggravation of scoliosis or sleep apnea, each for one patient.

Concerning the gonadotropic axis, whereas 9 out of 10 patients had hypogonadism, only half the patients of the entire cohort received sex-hormone therapy during adulthood. There was no difference depending on transition status or gender (54.5% vs 58.8%, *P* = 0.43 for male and female respectively). However, nearly ten times more patients in the transition group had sex-hormone therapy in childhood (Table 1).

Regarding the other endocrine functions, most patients were screened for the thyrotropic axis or had a measurement of cortisol level at 08:00 h. However, only 37.9% had a valid screening for the corticotropic axis, defined as performing a dynamic test if the cortisol level at 08:00 h was between 138 and 500 nmol/L. There was no difference depending on transition status (Table 1).

### Anthropometric, metabolic and psychiatric parameters depending on transition status

Table 2 presents the metabolic characteristics of the 95 patients according to transition status. Among the whole cohort, the mean BMI was close to 40 kg/m^2^, half the patients had grade III obesity and mean body fat was about 50%. A third of patients had type 2 diabetes and a fifth had HBP.

**Table 2:** Anthropometric, metabolic and psychiatric parameters according to transition status from pediatric to adult care.

	All (*n* = 95)	Transition (*n* = 31)	Without transition (*n* = 64)	*P*
Weight (kg)	96.1 ± 30.2 (39.9; 177.0)	80.0 ± 19.0	104.1 ± 31.6	**<0.01**
Height (cm)	155 ± 11 (120; 188)	157 ± 11	154 ± 11	0.23
BMI (kg/m^2^)	39.8 ± 12.1 (20.4; 77.6)	32.5 ± 7.8	43.4 ± 12.3	**<0.001**
BMI in normal range (%)	8 (8.4)	5 (16.1)	3 (4.7)	0.15
Overweight (%)	16 (16.8)	8 (25.8)	8 (12.5)	0.21
Obesity (grade I, II, or III) (%)*	69 (72.6)	18 (58.1)	51 (79.7)	**0.02**
Grade III obesity (%)	46 (48.4)	7 (22.6)	39 (60.9)	**<0.001**
Body composition				
Fat mass (%)	49.7 ± 6.5 (31.2; 60.0)	47.3 ± 6.8	51.0 ± 6.0	**0.01**
Fat mass (kg)	46.9 ± 17.6 (17.0; 90.3)	36.4 ± 10.5	52.4 ± 18.2	**<0.0001**
Lean body mass (kg)	44.3 ± 12.1 (20.4; 73.8)	38.7 ± 8.6	47.3 ± 12.6	**0.001**
Fat mass index (kg/m^2^)	19.7 ± 7.4 (6.6; 36.3)	15.0 ± 4.6	22.1 ± 7.5	**<0.0001**
Trunk fat mass/appendicular fat mass	1.0 ± 0.3 (0.1; 1.7)	0.8 ± 0.3	1.0 ± 0.2	**<0.01**
Metabolic parameters				
Diabetes (%)	27 (28.4)	6 (19.4)	21 (32.8)	0.22
Fasting blood glucose (mmol/L)	5.4 ± 2.7 (3.0; 20.9)	5.1 ± 2.7	5.5 ± 2.7	0.55
HbA1C in subjects with diabetes (%)	7.6 ± 2.0 (5.7; 14.6)	8.3 ± 1.9	7.5 ± 2.0	0.40
Fasting insulin (µIU/mL)	17.6 ± 25.7 (2.0; 185.0)	19.3 ± 35.6	16.7 ± 18.6	0.69
HOMA-IR	3.2 ± 3.2 (0.6; 17.7)	2.1 ± 1.6	3.7 ± 3.7	0.12
Total cholesterol (mmol/L)	4.5 ± 1.0 (0.8; 7.1)	4.4 ± 1.3	4.5 ± 0.9	0.47
HDL cholesterol (mmol/L)	1.1 ± 0.1 (0.1; 2.2)	1.2 ± 0.5	1.1 ± 0.3	0.08
LDL cholesterol (mmol/L)	2.8 ± 0.8 (0.4; 4.9)	2.6 ± 1.0	2.9 ± 0.7	0.20
Triglycerides (mmol/L)	1.5 ± 2.3 (0.3; 21.3)	1.0 ± 0.7	1.7 ± 2.8	0.22
AST (IU/L)	27 ± 10 (12; 66)	27 ± 12	27 ± 10	0.91
ALT (IU/L)	33 ± 25 (12; 127)	33 ± 30	33 ± 26	0.98
High blood pressure**	17 (17.9)	1 (3.2)	16 (25.0)	**0.02**
Systolic blood pressure (mmHg)	122 ± 14 (98; 171)	118 ± 12	124 ± 15	0.05
Diastolic blood pressure (mmHg)	70 ± 14 (32; 111)	68 ± 12	70 ± 14	0.45
Behaviour				
Skin picking	59 (62.1)	21 (67.7)	38 (59.4)	0.94
Neuroleptic treatment	45 (47.4)	10 (32.3)	35 (54.7)	0.05
Antidepressant treatment	29 (30.5)	4 (12.9)	25 (39.1)	**0.02**
Other psychotropic treatments	28 (29.5)	8 (25.8)	20 (31.3)	0.69
Hospitalization in psychiatry	28 (29.5)	6 (19.4)	22 (53.1)	0.16

*Fat mass index was calculated as fat mass in kg divided by height squared (kg/m^2^). **Systolic blood pressure >140 mmHg or diastolic blood pressure >90 mmHg.

Patients in the transition group had improved anthropometric parameters. They weighed significantly less and had a lower BMI and lower body fat, whether expressed as percentage of body weight, absolute FM or when taking into account height (FMI). They also had a more favorable body fat repartition than patients without transition as assessed by the trunk-to-appendicular FM ratio. They also had an improved metabolic profile, as only one patient in the transition group had HBP compared to 16 without transition (*P* = 0.02). The percentage of type 2 diabetes was lower in the transition group, but this difference did not reach significance.

Most patients of the whole cohort had behavioral or psychiatric disorders: 1/3 had a history of hospitalization in a psychiatric department during adolescence or adulthood, 2/3 displayed skin picking, half of the subjects received neuroleptic and 1/3 had antidepressant or another psychotropic treatment. However, fewer patients in the transition group had psychiatric disorders, as fewer received antidepressants or neuroleptics.

GH treatment during childhood is known to be associated with improved anthropometric and metabolic parameters in adulthood, as shown previously (17). We thus compared the transition and without transition groups according to GH treatment in childhood (Table 3). Patients who received transitional care in both the non-treated and treated groups weighed less and had a lower BMI, absolute FM and FMI than those who did not. Fewer patients who received transitional care in the GH-treated group received neuroleptic or antidepressant treatment.

**Table 3:** Anthropometric, metabolic, and psychiatric parameters of patients with Prader–Willi syndrome according to GH treatment in childhood and transition status.

	Group not treated by GH (*n* = 61)			Group treated by GH (*n* = 34)		
	Transition (*n* = 12)	No transition (*n* = 49)	*P*	Transition (*n* = 19)	No transition (*n* = 15)	*P*
Age (year)	25.7 ± 5.8	32.8 ± 9.8	**0.02**	23.2 ± 3.8	28.7 ± 6.3	**<0.01**
Female gender	7 (58.3)	30 (61.2)	1	10 (52.6)	4 (26.7)	0.24
Weight (kg)	83.3 ± 18.6	104.8 ± 32.9	**0.03**	78.0 ± 19.4	102.0 ± 27.8	**<0.01**
Height (cm)	152 ± 8	152 ± 12	0.96	160 ± 11	160 ± 9	0.88
BMI (kg/m^2^)	35.9 ± 7.9	44.7 ± 12.7	**0.03**	30.4 ± 7.1	39.2 ± 10.2	**<0.01**
Obesity	9 (75.0)	39 (79.6)	0.82	9 (47.4)	11 (73.3)	0.14
Grade III obesity (%)	5 (41.7)	31 (63.3)	0.22	2 (10.5)	8 (53.3)	**<0.01**
Body composition						
Fat mass (%)	51.0 ± 4.0	51.5 ± 5.6	0.80	44.9 ± 7.2	49.2 ± 6.9	0.11
Fat mass (kg)	39.5 ± 9.4	53.8 ± 19.0	**0.01**	34.5 ± 10.9	47.6 ± 14.7	**<0.01**
Lean body mass (kg)	37.9 ± 7.7	47.2 ± 13.0	**0.02**	39.2 ± 9.3	47.7 ± 11.9	**0.03**
Fat mass index (kg/m^2^)	17.0 ± 3.6	23.1 ± 7.5	**<0.01**	13.7 ± 4.8	18.7 ± 6.4	**0.02**
Trunk fat mass/appendicular fat mass	0.9 ± 0.2	1.0 ± 0.2	0.30	0.8 ± 0.3	1.0 ± 0.3	**0.02**
Metabolic parameters						
Type 2 diabetes	4 (33.3)	18 (36.7)	1	2 (10.5)	3 (15.8)	0.70
High blood pressure*	1 (8.3)	13 (26.5)	0.30	0	2 (13.3)	0.32
Behavior						
Neuroleptic treatment	7 (58.3)	27 (55.1)	1	3 (15.8)	8 (53.3)	**0.03**
Antidepressant treatment	3 (25.0)	16 (32.7)	0.80	1 (5.3)	9 (60.0)	**<0.001**
Hospitalization in psychiatry	2 (16.7)	16 (32.7)	0.41	4 (21.1)	6 (31.6)	0.26

*Systolic blood pressure >140 mmHg or diastolic blood pressure >90 mmHg. The fat mass index was calculated as fat mass in kg divided by height squared (kg/m^2^).

We performed multivariate analysis to evaluate the impact of transition status independently of GH treatment during childhood and/or adulthood. Transitional care remained significantly associated with BMI (*P* = 0.0006), percent body fat (*P* < 0.03), FMI (*P* < 0.0005) and neuroleptic treatment (*P* < 0.0007).

As age at first multidisciplinary assessment was significantly different in the two groups, we also did an analysis excluding patients who were >30 years old at first multidisciplinary assessment (*n* = 18). After exclusion of these patients, age at the multidisciplinary assessments was 19.9 ± 3.1 years in the transition group vs 22.1 ± 3.2 years in the without transition group (*P* < 0.01). BMI, percent body fat, FMI and antidepressant treatment remain lower in the transition group (32.5 ± 7.8 vs 44.3 ± 11.7 *P* < 0.0001 47.3 ± 6.8 vs 51.6 ± 5.8% *P* < 0.001, 15.0 ± 4.6 vs 23.1 ± 7.6 kg/m^2^ *P* < 0.0001 and 12.9 vs 43.5% *P* < 0.001). Moreover, patient in transition group had lower neuroleptic treatment (32.2 vs 56.5% *P* = 0.03).

### Social status in adulthood and patient-focused outcomes

The aim of the patient survey was to obtain the perspective of patients and their families on emerging medical and social problems, particularly in adulthood. Important social concerns emerged at the beginning of adulthood based on the responses of the 33 patients and their caregivers who answered the questionnaire. There were no significant differences depending on transition status. Most adults lived in the family home (*n* = 20/33), a situation perceived as a default choice in the absence of appropriate alternative solutions, eleven lived in a group home and only two lived alone. Most patients (*n* = 26/33) were educated in a special school for people with special disabilities, 6 were in middle and high school, and one was in university. At adulthood, 18 patients had no professional activity and 13 worked in a sheltered workplace. All patients received social benefits. Twenty-six patients had legal protection (guardianship or curatorship). The breakdown in social benefits was reported for five patients, among whom two were in the transition group. Patients and their families also described an increase in medical problems in adulthood, such as weight gain (*n* = 27/33), behavioral disorders (*n* = 23/33) or endocrine issues (*n* = 13/33).

## Discussion

Our study is the first to assess complementary aspects (endocrine, metabolic, psychiatric and social) of the medical transition process in PWS patients. We show that a coordinated care pathway with specialized pediatric care and organized transition from a pediatric hospital to a Reference Center with PWS is associated with improved endocrine, metabolic, anthropometric and psychiatric traits in adulthood. These results are in general agreement with previous literature describing transitional care for patients with other endocrine diseases. In this field, Bachelot *et al.* studied the impact of transitional care on patients with congenital adrenal hyperplasia and showed that transitional care was associated with regular medical care during adulthood (21). The data are also in line with the conclusions of a recent endocrinologist expert that highlighted the necessity of multidisciplinary collaboration across health sectors, the need for pediatric and adult endocrinologists to work together, early involvement of adolescents in their disease and involvement of parents in the transition process (11).

Only 35.8% of the patients in our cohort had received GH treatment during childhood. This is in contrast with the current guideline to start GH treatment during the first year of life, with no requirement of a GH stimulation test (13, 22). However, the low figure in our study is likely because GH therapy has only been approved for children with PWS without short stature or GHD in Europe since 2000, whereas patients in our study were born between 1956 and 1999. More patients of the transition group, who are younger, were treated. Thus, more future adolescents in transition will likely be treated than in our cohort.

Based on current recommendations, all patients should have had a GH stimulation test during transition or in adulthood prior to the continuation of GH treatment (19, 20). In our study, 50% of patients who had the test still had a GH deficiency, consistent with other previous studies (18, 23, 24, 25, 26). Furthermore, recent studies showed a beneficial effect of GH treatment in adulthood on cognitive ability (27, 28, 29), body composition (30, 31), physical activity (32), behavior (33) and bone structure (34). In addition, GH treatment does not appear to adversely affect the metabolic health profile (35). Thus, the maintenance of GH treatment in adulthood is justified in those who benefit from GH treatment in childhood. Surprisingly, transitional care was not associated with a significant difference in the assessment and/or treatment of the GH axis in adulthood, with few patients tested (16.8%) and/or treated (14.7%). The low rate of testing and treatment in our adult cohort was due to several reasons, the most frequent being behavioral disorders. Indeed, the surveys of the patient and their family confirmed the emergence of social, medical and psychiatric problems in adulthood. These problems negatively affected endocrine management of these patients. Thus, we recommend performing the GH stimulation test before transition from the pediatric department at the end of growth (14–15 years), as we suggested in a recent publication (11), because the passage to the adult setting is associated with other marked changes. We already recommended this in the National Health Plan on the PWS, but it takes time to change medical practice (36).

Ninety percent of our patients had hypogonadism, consistent with published series (2). However, only 21% in pediatric departments and 56% in adult department received sex-hormone therapy. It is well known that sex-hormone therapy improves the quality of life of patients with hypogonadism (37), and it has been shown that testosterone replacement therapy of PWS patients improved body composition and bone mineral density without adverse behavioral problems (38). The low rate of sex-hormone therapy in our study can be explained by the significant prevalence of pre-existing behavioral disorders in these patients. Indeed, aggressive behavior may cause the interruption of treatment in males. The fear of such aggressiveness may also limit the introduction of treatment by the practitioner or family. However, the introduction of sex-hormone therapy in these patients should always be considered, taking into account the benefits and risks, starting at 25% of the adult dose (39) to at least preserve bone mass.

The prevalence of central adrenal insufficiency in patients with PWS is not well known. In our study, only 37.9% of patients had correct screening of the corticotropic axis. Cortisol levels at 08:00 h were frequently measured, but the necessary dynamic tests were rarely performed. Central adrenal insufficiency should be systematically assessed in all patients. We recommend testing the corticotropic axis at the same time as the GH stimulation test by performing an insulin-hypoglycemic test. This should be done systematically, as the clinical signs of central adrenal insufficiency are non-specific, and the consequences of an acute adrenal crisis can be serious.

One important observation of our study is the link between effective transition and better health status in adulthood. The improvement in anthropometric and metabolic parameters is likely due to GH treatment, as more patients in the transition group received GH treatment during childhood. Indeed, it is well known that GH treatment in childhood improves both body composition (20, 40) and behavior (33). Nevertheless, the association between transition and an improved medical situation in our cohort of adults was found independent of GH treatment. These results highlight the need for a specific care pathway in this context of a complex disorder with close collaboration between pediatricians and adult physicians.

We must emphasize that the age at diagnosis in our study was relatively late and that the diagnosis of PWS is now performed during the neonatal period. This should improve the health of these patients during childhood and adolescence with possible better health outcomes in adulthood.

Our study shows that endocrine management in patients with PWS during the transition period is made difficult by multiple comorbidities, especially psychiatric disorders. Therefore, we recommend systematically performing a complete endocrine and neuropsychiatric assessment at the end of growth, in the pediatric unit before referring patients to an adult department, to improve endocrine management. Behavioral disorders can disrupt medical follow-up, which is directly linked to a poorer health status. To be effective, transition should not only focus on endocrine aspects, but also consider social, psychiatric and nutritional issues. In our cohort, pediatric follow-up with transition was associated with a lower prevalence of psychotropic treatment in adulthood especially among those who had benefited from GH treatment thus strengthening the potential link between transition, GH treatment and better mental health. A well-organized transition requires a multidisciplinary team to manage the disease as a whole, supervised by a specialist for adults with PWS, following early and structured care in a pediatric department. One of the missions of the French Reference Center, certified in 2004, is to ensure the patient a coordinated care pathway, with no disruption between childhood and adulthood, and to improve the care of PWS patients of all ages by pediatricians and adult physicians working with a multidisciplinary team (endocrinologist, dietician, psychologist and social worker).

In conclusion, our study shows that specialized pediatric care with transition to an adult multidisciplinary team as proposed in our PWS French Reference Center is associated with better anthropometric and metabolic outcomes, independently of GH treatment in patients with PWS. These patients need to be managed through a global approach, taking into account their medical, psychiatric, nutritional and social issues to properly assess and supplement their multiple endocrine dysfunctions. This can only be achieved through multidisciplinary teams able to coordinate care and establish the link with the social partners. We are thus developing several actions. We now conduct a multidisciplinary meeting prior to the first adult consultation, providing an opportunity to discuss not only medical aspects but also social dimensions (school and work orientation, functional abilities, legal protection, etc.). We have developed and are currently testing a transition booklet containing, in addition to medical aspects, paramedical correspondents and social and lifestyle information. Finally, we have developed a wider and comprehensive transition program for all endocrine diseases called ‘TransEnd’ (http://institut-e3m.aphp.fr/transend/) with the support of a patient pathway coordinator. This program has been recently implemented and is yet to be evaluated, but clinicians in charge of PWS patients have already noted better coordination of transitional care.

## Declaration of interest

The authors declare that there is no conflict of interest that could be perceived as prejudicing the impartiality of the research reported.

## Funding

This research did not receive any specific grant from any funding agency in the public, commercial or not-for-profit sector.
